# Ensuring that the voices of patients, care partners, and equity‐seeking communities are heard; a lived experience account

**DOI:** 10.1002/alz70858_104572

**Published:** 2025-12-26

**Authors:** Ngozi Faith Iroanyah

**Affiliations:** ^1^ Alzheimer Society of Ontario, Toronto, ON, Canada

## Abstract

As a caregiver, EDI advocate, Alzheimer Society Director, and researcher: I am uniquely positioned to participate in this session and ensuring that the voices of patients, care partners, and equity‐seeking communities are heard.

As the Director of Health Equity and Access for the Alzheimer Society of Ontario, I have designed, led, and supported the development of health equity initiatives to support our provincial federation of 26 local Alzheimer's societies. I provide strategic support and advice on health equity programs; develop and implement Knowledge Translation and Exchange products and community engagement strategies; and collect data and conduct research. I have built strong, collaborative relationships with community partners across the province. I sit on the Board of Directors for the Black Health Alliance, a non‐profit that supports, advocates, and develops programs to enhance and protect the health status of Black Canadians.

I am a PhD scholar at York University in Health Policy & Equity and have sizeable experience and training in health policy research, especially dementia care through the perspective of people with lived experience and/or from marginalised communities. I received the Doctoral Personnel Award for Black Scholars from the Heart and Stroke Foundation of Ontario as recognition for my work including national and international articles and presentations on dementia, diversity, and community engagement.

I regularly conduct media presentations, and public speaking engagements in various equity deserving communities, discussing dementia from a community centered lens, helping to destigmatize the disease and build health literacy and resilience.

As the primary guardian for my father for over 17 years, I have been heavily involved with the entire dementia journey: first identification, early diagnosis, and accessing supports and health services. My experience as a caregiver and advocate has given me insight into how care is delivered, and received (from a cultural perspective), and where opportunities for growth and change exist.

By leveraging these experiences, I am able to participate and fulsomely contribute to discussions on dementia, research inclusivity, caregiving, and systems level responses to the needs of people living with dementia and their care partners.

